# Multiple Imputation in Survival Models: Applied on Breast Cancer Data

**Published:** 2011-08-01

**Authors:** M R Baneshi, A R Talei

**Affiliations:** 1 Department of Biostatistics and Epidemiology, Kerman University of Medical Sciences, Kerman, Iran; 2 Department of Surgery, Shiraz University of Medical Sciences, Shiraz, Iran

**Keywords:** Prognostic model, Missing data, Multiple imputation, Breast cancer

## Abstract

**Background:**

Missing data is a common problem in cancer research. While simple methods such as completecase (C-C) analysis are commonly employed for handling this problem, several studies have shown that these methods led to biased estimates. We aim to address the methodological issues in development of a prognostic model with missing data.

**Methods:**

Three hundred and ten breast cancer patients were enrolled. At first, patients with missing data on any of four candidate variables were omitted. Secondly, missing data were imputed 10 times. Cox regression model was fitted to the C-C and imputed data. Results were compared in terms of variables retained in the model, discrimination ability, and goodness of fit.

**Results:**

Some variables lost their effect in complete-case analysis, due to loss in power, but reached significance level after imputation of missing data. Discrimination ability and goodness of fit of imputed data sets model was higher than that of complete-case model (C-index 76% versus 72%; Likelihood Ratio Test 51.19 versus 32.44).

**Conclusion:**

Our findings showed inappropriateness of ad hoc complete-case analysis. This approach led to loss in power and imprecise estimates. Application of multiple imputation techniques to avid such problems is recommended.

## Introduction

Prognostic models combine key patient characteristics (risk factors) to predict clinical outcomes such as recurrence of cancer. These models are excellent tools to investigate the contribution of variables to disease course, and to select the appropriate treatment paths.[1]

However, if in development of model, one ignores model assumptions, results might be misleading.[2][3] One of the issues that challenge the modelling practice is incomplete data. A problem in survival analysis occurs when data are missing on risk factors.[4] The traditional response to this problem is to exclude individuals with incomplete data on any prognostic factors from analysis (Known as Complete-Case Analysis (C-C analysis).[4]

However, exclusion of missing data leads to attrition in sample size which will diminish precision of estimates and can lead to biased estimates.[5][6] Therefore, appropriate methods should be applied to impute missing data. Methodological developments in the filed of analysis of missing data offers a lot to modelling. Advanced likelihood-based methods can be applied to use partially observed data so as to predict missing values. This preserves attrition in sample size and avoids biased estimates.

There are lots of methods to tackle the problem of missing data. The main aim of this paper is to highlight the methodological issues in development of a prognostic model in presence of missing data. Here we only focused on the Multivariable Imputation via Chained Equations (MICE) method. The MICE is a flexible method which has the capability to deal with all forms of variables (continuous, categorical, and binary), and can be used in regression settings. Methods were applied analysing a breast cancer data set. To show the power of the MICE method in recovery of information, prognostic models were developed using complete data as well as imputed data sets.

## Materials and Methods

From 1994 to 2003, the information of 310 breast cancer patients in Shiraz (located in southern Iran) with a median follow-up of 2.5 years, were collected from Hospital-based Cancer Registry of Nemazee Hospital (affiliated to Shiraz University of Medical Sciences). The end point of the study was death. At the end of the study, there had been 56 deaths.

Variables offered to the multifactorial models were those showed to have univariate predictive ability (tumor stage with 3 levels (early, locally advanced, and advanced), tumor grade with 3 levels (1, 2 and 3), history of benign breast disease (positive versus negative), and age at diagnosis (≤47 versus >47).[7] The data set do not include personal information such as name, address, or phone number of patients.

For analyzing of data, Kaplan-Meier and Log-rank tests were used to compare the survival curves in different groups. Linear Cox model was then applied to develop the multifactorial regression models and to estimate Hazard Ratios (HR).[8] Two models were used using Complete-Case (C-C) data and imputed data sets. To impute the missing data, Multivariable Imputation via Chained Equations were applied (MICE model).

In the C-C model, patients with missing data on any of 4 variables selected were excluded. Cox regression model in conjunction with ENTER variable selection method was then fitted to patients with available data on all 4 candidate risk factors. A final risk score was calculated by multiplying variables into the estimated regression coefficient. Tertiles of the risk score estimated were applied as cut off to categorise patients into low (L), intermediate (I), and high (H) risk groups.

The MICE method is a probabilistic approach. The usual practice to reflect the uncertainty about the true values of the missing data, is to replace each missing value by 10 values leading to 10 imputed data sets.[9][10] The process of the MICE method is described below:

To identify the mechanism of missing data, an indicator variable for each of variables which had missing data was created. For example, indicator variable for stage variable shows whether patient had missing value or not. Patients with available data get a value of 1 while others get 0. The association between this indicator variable, showing stage missing and rest of variables were assessed applying Chi-Square test. When the missingness depends on observed variables mechanism, it is called Missing At Random (MAR).

It has been suggested that, for best imputation, the outcome variable should be included in the imputation model.[11] Therefore, patients' outcome and set of four risk factors were used in the MICE algorithm.

Polytomous and logistic regression were used to impute missing data for categorical (stage and grade) and binary data (age and benign disease history) respectively.

The MICE method involves no distributional assumption and can be used to impute missing data for continuous, categorical, and binary variables. To impute missing value on a variable which include missing data, say X j , a regression model relates X j to other variables in the imputation model. This regression model is then used to create imputed values by drawing from the posterior predictive distribution. Each predictor with missing values is considered in turn using the current imputed values for each of the other predictors.[12] The iteration process ends when all variables had been updated technical details are given in Appendix.[13][14] This entire process was repeated and the imputed values which are created at the 5th round were used as the first imputed data set. The whole processes were repeated 10 times to replace each missing data by 10 values, thus creating 10 data sets.[12] The standard algorithm imputes each incomplete column in the data from left to right. It is known that this issue (i.e. order of the variables) is essentially irrelevant to the results.

The creation of 10 data sets means there is a requirement for 10 modelling analyses, one for each data set, and there will therefore be 10 different estimates for each parameter. Estimates derived from imputed data sets (the coefficients and standard errors) therefore, need to be combined and this was achieved applying Rubin’s rule.[14] The final regression coefficient is simply the average of coefficients across imputed data sets.[14] In estimation of standard errors, both between and within imputation variations should be taken into account technical details are given in Appendix.

Hazard Ratios (HR) and corresponding 95% Confidence Intervals (C.I.) were calculated from regres sion coefficients and standard errors that have been imputed across multiply imputed data sets.

A risk score was calculated for each of 10 imputed data sets. For each patient, a single averaged risk score was calculated by averaging her estimated risk scores from each of the 10 imputed data sets.

In risk stratification studies, it is important to create risk groups where patients in each group are equally likely to develop the outcome.[15] Discrimination refers to the ability to separate patients with different responses15 and is measured using Harrell’s Cindex (concordance index) which is a generalization of Area Under Curve (AUC).[16][17] The C-index is interpreted as correct ordering in the sense that comparing risk predictions for two patients, risk calculated for whom developed the disease is higher than the other one. This statistic varies between 0.5 and 1 where values near 1 indicate high discrimination power. However, if performance is assessed on the same sample as used for model development, then performance will be overestimated. Therefore, bootstrap procedure was applied and bias-corrected Cindices were reported.[18]

For all models, we will report Likelihood Ratio Test (LRT) which indicates how well the model fits the data. A series of packages which work under R software (version 2.5.1) were used.19 Missing data were imputed using MICE package. Estimated regression coefficients and standard errors were combined across imputed data sets using Mitools library. Performance of models (discrimination and predictive ability) was assessed using Design library. K-M curves are plotted using SPSS software.

## Results

Information for age variable was available for all patients. The variables nodal status and grade involved about 20% missing rate (20.3% and 20.6% respectively). Corresponding figure for 'history of benign disease' was 15.2%. However, after exclusion of missing data on all four variables, 35% of data were lost. Totally, 203 cases (65%) had data available on all 4 variables. Almost all patients with missing data were those survived. Out of 56 deaths only 2 ones were lost in complete-case analysis. We first examined missing data mechanism (Table 1). As shown, patient's status, grade and history of benign disease can predict missingness on stage variable. Patients' status and history of benign disease were predictors of grade missing. Furthermore, patients' status and age at diagnosis were predictors of benign disease variable. This confirms that data had a Missing At Random (MAR) mechanism.

**Table 1:** Investigation of the association between variables' missingness and the rest of variables^a^

**Missing indicator**	**Status**	**Stage**	**Grade**	**Benign disease**	**Age**
Stage	+		+	+	-
Grade	+	-		+	-
Benign disease	+	-	-		+

^a^ +: association between missing indicator and variable, -: lack of association between missing indicator and variable

Estimated Hazard ratios (HR) with 95% Confidence Intervals (C.I.), corresponding to complete-case and imputed data sets are given in Table 2. Age at diagnosis and history of benign disease were not significant in complete-case model, due to attrition in sample size and inevitable loss in power of model. Furthermore, risk of death for patients with stage 3 relative to those with stage 1 was not significant in complete-case model. After imputing missing data, both of these variables (age at diagnosis and family history of benign disease) were retained in the model. In addition, HR for cases with stage 3 relative to stage 1 reached a significance level.

**Table 2:** Comparison of estimated HRs (95% C.I.s) corresponding to analysis of complete-case and imputed data sets a

**Variable**	**Level**	**Complete-case model (N=203, D=54)**		**Imputed data sets model (N=310, D=56)**	
		**HR (95% C.I.)**	**P value**	**HR (95% C.I.)**	**P value**
Stage	1	1		1	
	2	2.89 (1.52, 5.51)	0.001	3.13 (1.64, 5.97)	< 0.001
	3	1.94 (0.81, 4.63)	0.13	2.53 (1.05, 6.12)	0.03
Grade	1	1		1	1
	2	2.46 (1.61, 5.23)	0.02	2.46 (1.15, 5.24)	0.02
	3	1.33 (0.58, 3.04)	0.50	1.52 (0.65, 3.60)	0.34
Age	< 48 years	1		1	1
	≥ 48 years	1.75 (0.91, 3.38)	0.10	1.92 (1.01, 3.65)	0.04
Benign	No	1		1	
	Yes	1.91 (1.04, 3.49)	0.04	2.32 (1.24, 4.33)	0.01
Performance of models					
C-index		72%		76%	
Likelihood ratio test		32.44		51.19	

^a^ HR: Hazard Ratio, C.I.: Confidence Interval, N: Sample size, D: Number of deaths

Comparing performance of models, imputation of missing data led to 4 percentages point improvement in discrimination ability of model (76% for the MICE versus 72% for C-C data). Furthermore improvement in model goodness of fit was seen (51.19 versus 32.44).

## Discussion

Missing data are a common problem in medical and epidemiological data sets. Exclusion of missing data leads to loss of power. In results presented, some variables lost their significant effect in complete-case analysis. For example, stage of disease is known as one of the most important prognostic variables.[20][21] However, this variable did not reach to the significant level in the C-C model.

On the other had, in order to protect against chance effects dues to imputation, we imputed 10 data sets. This protection was to be felt worth the inconvenience of having to average risk scores across 10 final models. Once missing data were imputed, power was increased and variables lost their effect in complete-case model (such as stage of disease) and reached a significance level.

We also showed that our data had a Missing At Random (MAR) mechanism. This means that missing data depends on other patients' characteristics and therefore can be well imputed using multiple imputation methods. We should emphasize that our main goal was to illustrate the process of development of a prognostic model when missing data exist. To achieve this, we simply used a breast cancer data set in southern Iran as a prevalent cancer in this region set as an example.[22][23] Discussion of risk factors of breast cancer is beyond the scope of this paper and were previously reported.[22][23] This issue has been addressed here.[20][21]

It should be noted that, when missing rate is low, results of C-C model, in terms of variables retaining in the final model, might be similar to that of MICE. Asia Pacific Cohort Studies Collaborators (APCSC) collects data to determine Coronary Heart Disease (CHD) risk factor. Ability of multiple imputation and complete-case analysis to handle the missing data on a single variable (cholesterol) in 26 studies was compared. [24] Cholesterol missing rate varied from 0% to 69%. In 22 studies where cholesterol value was not available for about 10% of subjects both methods gave similar results. On the other had, in four studies with missing rate between10% to 60%, clear difference was seen between models. It has also been commented that with more than 60% missing rate, the MICE model might not provide accurate estimates.[24]

However, we believe that a low rate of missing data on each variable might cause serious problems in multivariate modelling when patients with missing data on different variables are not the same because this might substantially reduce the number of complete cases available for analysis, and increase the chance of bias due to excluded cases.

We developed the multifactorial models in conjunction with ENTER variable selection method. When Backward Elimination (B.E.) variable selection is hired, a series of iterative steps are required to exclude variables which do not contribute significantly to the model. If a single multifactorial model was developed, then application of B.E. is straightforward. However, when there are 10 imputed data sets, B.E. will not directly be feasible. In an iterative process, at each step, the results were aggregated across the 10 data sets, and the variable with the highest P-value (exceeding 0.05) was removed. Another set of 10 models were fitted with remaining variables, results were aggregated, and P-value assessed for a variable to drop (if p-value >0.05). The whole process continued until all variables remained significant.[12][13]

Before development of multifactorial models, we dichotomised the variable age at 48 because we showed that dichotomised version of this variable, in comparison with continuous form, improved the quality of the model.[25] Therefore in this study, only information on 2 binary and 2 categorical variables were analysed. When continuous data are available, Predictive Mean Matching (PMM) technique can be employed. In the PMM method, the complete-case whose value is closest to the imputed value is chosen. It takes the observation from the complete-case as the imputed value.

Our work involved several limitations. We used a data set contained only four variables. Therefore, impact of number of variables offered to the multifactorial model was not investigated. Furthermore, we only compared performance of the C-C and the MICE at 35% missing rate and under MAR mechanism. It is known that performance of models depends to a great extent to mechanism of missing data, rate of missing data, method of imputation of missing data, and sample size.[26][27][28][29] Our work was simply a case study to explain the methodological issues in the application of the MICE method, and its art in recovery of information.

Therefore, it is needed to design future studies so as to compare the performance of imputation models under different scenarios (i.e. by changing the sample size, missingness mechanism, missing rate, and method of imputation). We already showed that the C-C model decreases the power and the MICE method recovers the data. However, at this stage, due to limitations listed above, we cannot provide a specific guideline on how best to tackle the problem of missing data because there are lots of approaches to deal with missing data.[30] It has been shown that under special circumstances, alternative methods with easier methodology (such as replacement of missing data by mean of observed values) might provide comparable estimates. Application and comparison of alternative imputation methods were beyond the scope of this paper and will be published elsewhere.

Results presented showed how exclusion of missing data affect the composition of the model. Application of ad hoc methods such as complete case analysis is hugely criticised.[31][32] When complete-case gives results comparable to that of the MICE method, a gold standard such as the MICE method is required to compare results with other simpler methods. Therefore, application of such methods is highly recommended.
